# Pazopanib as Treatment Option for Pseudomyogenic Hemangioendothelioma: A Case Report

**DOI:** 10.7759/cureus.25250

**Published:** 2022-05-23

**Authors:** Ali Alhanash, Mohamed Aseafan, Jean Atallah

**Affiliations:** 1 Oncology, King Faisal Specialist Hospital & Research Centre, Ryiadh, SAU; 2 Section of Medical Oncology, Department of Internal Medicine, Security Forces Hospital Program, Riyadh, SAU; 3 Oncology, King Faisal Specialist Hospital & Research Centre, Riyadh, SAU

**Keywords:** sarcoma, tki, epithelioid sarcoma-like hemangioendothelioma, pseudomyogenic hamangioendothelioma, pazopanib

## Abstract

Pseudomyogenic hemangioendothelioma (PHE) also known as epithelioid sarcoma-like hemangioendothelioma (ES-H) is a vascular lesion of intermediate grade biologically behaving between benign hemangioma and malignant angiosarcoma. We present a 35-year-old male with an unremarkable medical history, who was referred to a sarcoma clinic complaining of right heel pain with equinus deformity and a mass in his right lower limb for 6 months. Biopsy was performed and reported as Pseudomyogenic Hemangioendothelioma. The patient was started on pazopanib with a favorable clinical and radiological response. Long-term follow-up is still needed, however further studies are vital to clarify the role of Tyrosine Kinase Inhibitor therapy.

## Introduction

Soft tissue sarcoma (STS) is a heterogeneous group of tumors with over one hundred histologic subtypes that requires a pathologist specialized in sarcoma for diagnosis [1]. Pseudomyogenic hemangioendothelioma (PHE), also known as epithelioid sarcoma-like hemangioendothelioma (ES-H), was first described in 2013 by WHO. It is a vascular tumor biologically behaving like benign hemangioma and malignant angiosarcoma and rarely metastasizes [2,3]. In the fifth edition of WHO classifications of bone and soft tissue tumors published in 2020, PHE was further characterized by a balanced t(7;19) translocation causing a SERPINE1-FOSB fusion [4].

PHE predominantly affects young adults at age of 20-40 years with a male to female ratio of 4.6:1 with muscle (34%) being the most commonly affected tissue followed by dermis (31%), subcutaneous tissue (20%), and bone (14%). Most commonly, it affects the lower extremity in deep soft tissues accounting for 60% of the cases found in the literature [5,6]. Studies on PHE management are still lacking, particularly in patient subgroups [7]. In the present case report, we report the clinical activity of pazopanib in a Pseudomyogenic hemangioendothelioma patient.

## Case presentation

We present a 35-year-old male with an unremarkable medical history who was referred to a sarcoma clinic complaining of right heel pain with equinus deformity and a mass in his right lower limb for 6 months. He underwent an excisional biopsy in a community hospital that came suspicious for neoplasia and inconclusive. On examination, his right foot showed equinus deformity along with multiple skin nodules in the tibia. The neurovascular exam was unremarkable.

**Figure 1:**
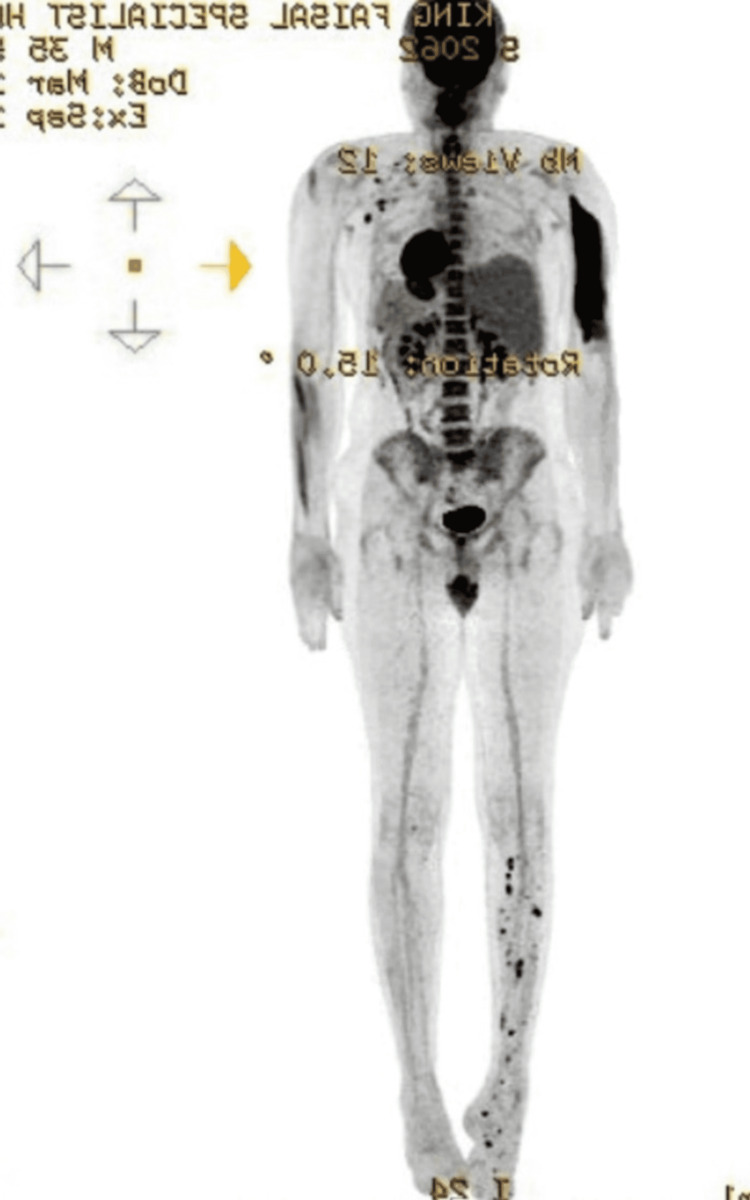
FDG PET-CT whole body FDG-avidity in the deep subcutaneous/intramuscular foci involving the right lower limb

Initial diagnostic MRI revealed nonspecific multiple subcutaneous and muscular enhancing nodules associated with osteopenia involving the right tibia and foot. PET CT done in September 2021 showed FDG-avidity in the deep subcutaneous/intramuscular foci involving the right lower limb. No suspicious FDG-avid lesions were seen elsewhere (Figure 1).

Biopsy was repeated at our center and reported as Pseudomyogenic Hemangioendothelioma. Immunohistochemistry panel revealed: SMA (-), DESMIN (-), S100 (-), CD34 (-), AE1/AE3 (+), CD31 (+); IHC staining for FOSB was not available in our laboratory.

**Figure 2:**
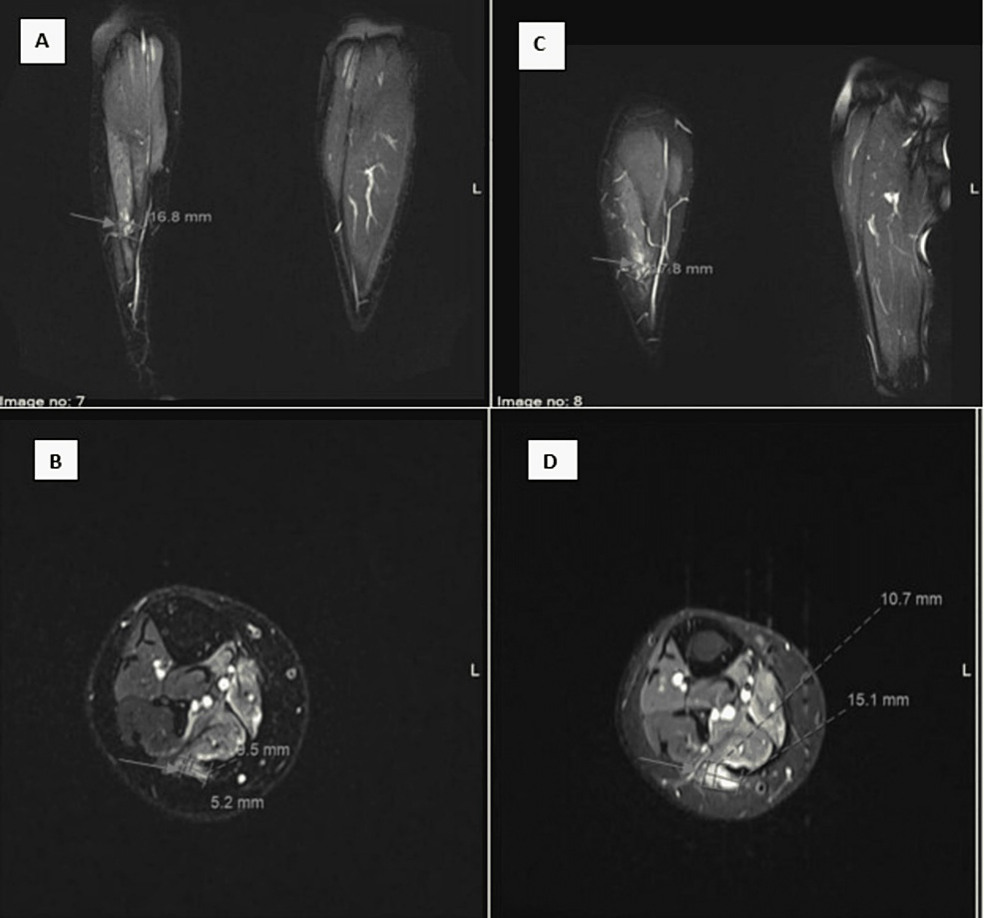
MRI right lower limb pre/post pazopanib A&B MRI right lower limb post pazopanib C&D MRI right lower limb pre pazopanib

The case was discussed at the multidisciplinary tumor board and the consensus was to go for systemic therapy. The patient was started on pazopanib 800 mg daily on October first. Subsequently, it was decreased to 600 mg due to GI intolerance with good clinical response in terms of decreased pain. Patient had imaging re-evaluation with leg MRI done in December 2021 that demonstrated a mild interval decrease in the size of the previously described multiple scattered subcutaneous and intramuscular enhancing nodules of the right leg and foot with the largest lesion in the posterolateral aspect of the lateral head gastrocnemius muscle currently measuring 0.5 x 1 x 1.7 cm (previously was 1.1 x 1.5 x 1.8 cm) in maximum AP, transverse and craniocaudal dimensions, respectively (Figure 2). No new soft tissue lesion is seen. 

Another dose reduction in pazopanib was required to 400 mg due to significant fatigue and anorexia. He was also noted to develop generalized skin and hair hypopigmentation. In March 2022, MRI to assess response was done again showing disease stability.

## Discussion

There are few reports on PHE. In 2017, Pradhan et al. have reported 8 patients with males representing 7 out of 8 patients (87.5%) and a mean age at presentation of 29 years. In this case series, the mean tumor size was 1.9cm ranging between 0.3 and 5.5 cm. The most common site of involvement was the leg in 6 patients (75%) with the remaining two cases occurring on the arm [8]. Half of the cases involved the bone, while the other half were restricted to the superficial and deep soft tissue (25% and 25% respectively). At presentation, a total of five cases (62.5%) showed multifocal disease. The second case series reported in 2011 by Hornick and Fletcher describe fifty patients with a male predominance (82%) and mean age of 31 years at diagnosis [6]. In more than half of the cases (54%), the primary site was in the leg, followed by 24% on the arm, 18% on the trunk, and 4% on the neck. Tumor involved the dermis and subcutaneous tissue in 52%, followed by skeletal muscle 34%, and bone 14%. Again, 66% showed multifocal disease at presentation. Our patient sex, age, and tumor characteristics were consistent with previously published data.

Since Pseudomyogenic (epithelioid sarcoma-like) Hemangioendothelioma (PHE) was introduced as a new distinguished entity of vascular endothelial neoplasm in 2013, no standard of care or treatment was established for such cases. The literature reports various treatment options such as local control (surgical resection, and cryotherapy) and systemic therapy (chemotherapy, Mammalian target of rapamycin (mTOR) inhibitors, Tyrosine Kinase Inhibitor (TKI), bone resorptive agent, and Vascular endothelial growth factor (VEGF) inhibitor like bevacizumab) [9].

In a report of 58 clinical cases of PHE with bone and soft tissue involvement, 69% underwent local treatment with excision or curettage, whereas 20.7% had amputations. Seven patients were treated with mTOR inhibitors and 4 patients with anti-resorptive treatments in those with multifocal lesions not amenable to local treatment [10]. Cytotoxic chemotherapy and mTOR have showed clinical and radiological benefit [11]. Gemcitabine + docetaxel regimen was used in one patient with favorable response. However, it did not yield tumor shrinkage in another patient [11]. DNA sequencing of one patient revealed a pathogenic mutation of the tuberous sclerosis 1 (TSC1); everolimus was administered for this patient resulting in mild shrinkage of PHE metastases [11].

Telatinib was the first investigational TKI tried in PHE In 2018 [12]. In that study, the patient with extensive PHE was given telatinib, and showed a durable complete remission. The in vitro model for PHE showed that telatinib affected the self-regulated expression of the fusion gene throughout VEGF- and PDGF-receptor signaling and regulating SERPINE1 [12]. Our patient has received pazopanib which is a multi-target TKI and demonstrated clinical benefit with disease regression that is durable with 6 months of follow-up. Similar to Telatinib, Pazopanib is a promising treatment option for PHE that warrants further study in the future. Pazopanib is showing promise in this rare disease and its role should be explored further in this tumor type.

## Conclusions

With the inadequate knowledge of the pathogenetic mechanisms and the lack of randomized controlled trials, treatment of PHE remains unclear, and its indolent behavior makes extensive surgery with amputation more aggressive than needed. However, further studies with more patients are required to clarify the role of pazopanib in PHE.
